# Ablación de fibrilación auricular en paciente con dispositivo de oclusión septal interatrial. Reporte de un caso.

**DOI:** 10.47487/apcyccv.v4i2.288

**Published:** 2023-06-30

**Authors:** Renee Montesinos-Segura, Diego Davila-Flores, Fernando Quevedo-Candela, Mario Cabrera-Saldaña, Pío Zelaya-Castro, Richard Soto-Becerra

**Affiliations:** 1 Instituto Nacional Cardiovascular, INCOR-EsSalud, Lima, Perú. Instituto Nacional Cardiovascular INCOR-EsSalud Lima Perú; 2 Unidad de arritmias, Instituto Nacional Cardiovascular, INCOR-EsSalud, Lima, Perú. Unidad de arritmias Instituto Nacional Cardiovascular INCOR-EsSalud Lima Perú

**Keywords:** Defectos del Tabique Interatrial, Dispositivo Oclusor Septal, Fibrilación Auricular, Ablación por Catéter, Ecocardiografía, Heart Septal Defects, Atrial, Septal Occluder Device, Atrial Fibrillation, Catheter Ablation, Echocardiography

## Abstract

Los dispositivos de oclusión del septo interatrial dificultan el abordaje transeptal para la ablación de la fibrilación auricular, por lo que es necesario contar con métodos de imagen que guíen con seguridad la punción transeptal, como la ecocardiografía intracardíaca (EIC). Se describe el caso de un paciente de 49 años con fibrilación auricular paroxística sintomática, refractaria a fármacos antiarrítmicos, portador de un dispositivo de oclusión del septo interatrial, con intento previo de ablación fallido. La ablación de la fibrilación auricular se realizó con el sistema de mapeo 3D Carto V7, la punción transeptal fue guiada por EIC y el procedimiento fue exitoso. Este reporte de caso resalta la importancia de la multimodalidad en imágenes, con el fin de se logre una punción transeptal exitosa y eficaz para llevar a cabo la ablación de fibrilación auricular en pacientes portadores de dispositivos de oclusión septal interatrial.

## Abstract (en)

Interatrial septal occlusion devices hinder the transseptal approach for atrial fibrillation ablation, making it necessary to have imaging methods that safely guide transseptal puncture, such as intracardiac echocardiography (ICE). We describe the case of a 49-year-old patient with symptomatic paroxysmal atrial fibrillation, refractory to antiarrhythmic drugs, wearing an interatrial septal occlusion device, with a previous unsuccessful ablation attempt. Atrial fibrillation ablation was performed using the Carto V7 3D mapping system, the transseptal puncture was guided by ICE, and the procedure was successful. This case report highlights the importance of multimodality imaging to achieve successful and effective transseptal puncture for atrial fibrillation ablation in patients with interatrial septal occlusion devices.

## Introducción

La estrategia del control del ritmo es considerada ideal para el manejo de la fibrilación auiruclar (FA) sintomática y recurrente ^1^; sin embargo, la evidencia sobre el uso de antiarrítmicos en pacientes con cardiopatías congénitas (CC), como los defectos del septum interatrial, es limitada, y presenta baja eficacia y efectos adversos ^2^. En consecuencia, la ablación mediante radiofrecuencia ha surgido como una alternativa cada vez más utilizada, principalmente en pacientes refractarios al manejo médico ^1^. En los pacientes con CC y FA es frecuente observar limitaciones en los accesos venosos y abordaje transeptales debido a las cirugías o procedimientos previamente realizados, por lo cual se requiere de un conocimiento profundo de la anatomía cardiaca obtenida por ecocardiografía, resonancia magnética y/o tomografía computarizada ^2^. La punción transeptal en pacientes con dispositivo de oclusión septal interatrial presenta dificultades técnicas; sin embargo, es factible en la mayoría de los casos ^3^. El acceso se obtiene atravesando el tabique nativo o a través del dispositivo ^3^. Por lo desafiante de este procedimiento se han reportado pocos casos de ablación de FA en pacientes con oclusores del septo interatrial. Presentamos el reporte del primer caso de ablación de FA en un paciente con un dispositivo oclusor del septo interatrial en el Instituto Nacional Cardiovascular «Carlos Alberto Peschiera Carrillo» (INCOR).

## Reporte de Caso

**Figura 1:**
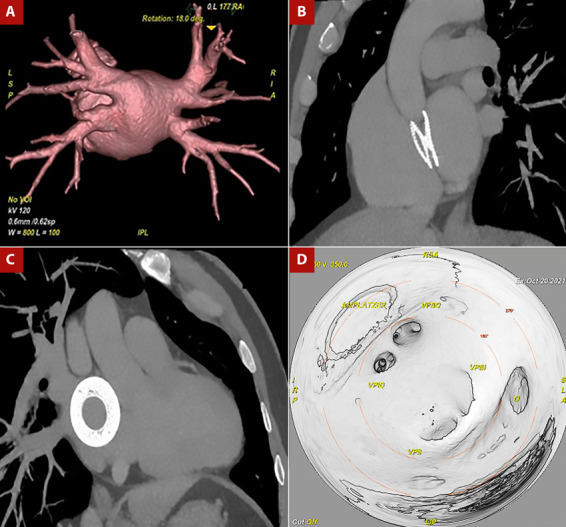
Angiotomografía cardiaca. A. Presencia de cuatro venas pulmonares que drenan hacia la aurícula izquierda, cada una con un ostium propio. B-C. Presencia de dispositivo oclusor a nivel del septum interauricular, con remanente en la región inferoposterior por donde se realizó la punción transeptal. D. Vista endoscópica de aurícula izquierda donde se evidencia el septum interauricular con un dispositivo oclusor, y el ostium de las venas pulmonares sin alteraciones de la anatomía.

VPSD: vena pulmonar superior derecha; VPID: vena pulmonar inferior derecha; VPSI: vena pulmonar superior izquierda; VPII: vena pulmonar inferior izquierda.

**Figura 2:**
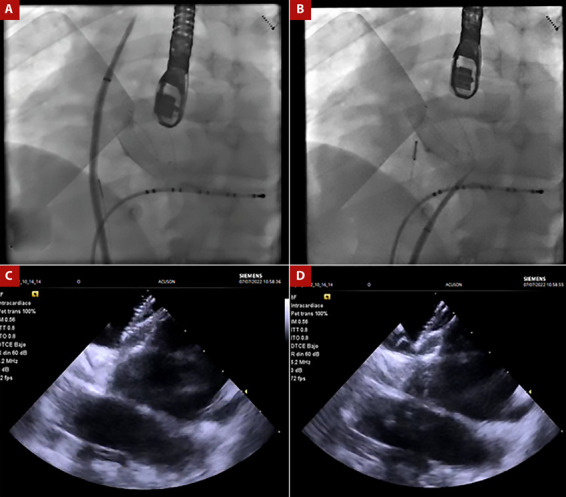
A. Vista fluoroscópica en proyección oblicua anterior izquierda donde se observa el introductor largo con la aguja de punción transeptal ubicados en la vena cava superior B. Presencia de introductor largo y la aguja transeptal a nivel del borde posteroinferior del dispositivo oclusor en el septum interauricular remanente. C. Imagen de ecografía intracardiaca que evidencia septum interauricular remanente en región posteroinferior y dispositivo oclusor. D Presencia de imagen en «tienda de campaña» en septum interauricular remanente cuando la aguja está a punto de atravesar hacia la aurícula izquierda.

Paciente varón de 49 años, con antecedente de cierre de una comunicación interatrial tipo ostium secundum el año 2017, con un dispositivo oclusor del septum interatrial Amplatzer (Nit occlud N° 26). Al examen físico sin hallazgos relevantes. El paciente cursó con palpitaciones taquicárdicas asociadas a registro electrocardiográfico de fibrilación auricular paroxística y fluter atrial típico, siendo muy sintomático y refractario a terapia antiarrítmica, incluso presentó efectos adversos asociado a amiodarona como el hipertiroidismo. Adicionalmente, tuvo un intento frustro de ablación de FA por punción transeptal fallida guiada por ecocardiograma transesofágico en el 2019. Por tal motivo se realizaron estudios anatómicos previos al procedimiento cuyos hallazgos se describen en la Figura 1. Realizamos la ablación de fibrilación auricular con radiofrecuencia empleando el sistema de mapeo 3D Carto V7 (Biosense - Webster, Diamond Bar, California), mediante abordaje transeptal guiado por EIC, por lo que posicionamos la sonda de EIC Acunav ® en la aurícula derecha para visualizar el septum interatrial. Luego pasamos el introductor largo SL1 (Swartch Left, Abbot) posicionándolo en vena cava superior; posteriormente avanzamos la aguja de punción transeptal (BRK, St. Jude) (Figura 2). Inicialmente se guio la punción transeptal por fluoroscopia observando doble salto y ubicándonos en la parte caudal y anterior del Amplatzer; después, guiados por la EIC alcanzamos a evidenciar una imagen de «tienda de campaña» sobre el septum interatrial (SIA) remanente, y observamos en tiempo real el avance de la aguja sobre el introductor largo y el septum interatrial alcanzando la aurícula izquierda. 

**Figura 3:**
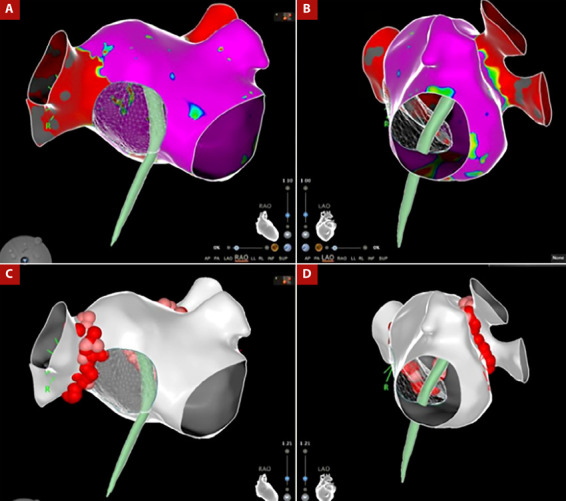
A-B. Reconstrucción 3D de la aurícula izquierda con mapa de voltaje, el cual muestra zonas de bajo voltaje en las venas pulmonares izquierdas que expresan aislamiento eléctrico. C-D Reconstrucción anatómica 3D de aurícula izquierda con imagen de la punción transeptal, donde se observan los puntos de ablación por radiofrecuencia en la región antral circunferencial de las cuatro venas pulmonares.

Debido a la no progresión de la vaina sobre el septum interatrial remanente, fue necesario intercambiar la aguja de punción transeptal con una guía 0,035” pasándola hacia las venas pulmonares izquierdas y con este soporte avanzamos el introductor largo hacia la aurícula izquierda. Debido a la dificultad del abordaje decidimos realizar el procedimiento con solo una punción transeptal. Pasamos el catéter de mapeo de alta densidad PENTARAY (Biosense - Webster, Diamond Bar, California) para realizar la reconstrucción anatómica tridimensional de la aurícula izquierda y las cuatro venas pulmonares (VPs), seguido del paso del catéter de ablación curva D con sensor de fuerza de contacto THERMOCOOL SMART TOUCH - SF NAV (Biosense - Webster, Diamond Bar, California), con lo que realizamos el aislamiento antral circunferencial de las cuatro VPs, aplicamos radiofrecuencia punto a punto empleando el *software* VISITAG SURPOINT (Biosense - Webster, Diamond Bar, California) alcanzado un índice de ablación de 400 en pared posterior y 550 en pared anterior (Figura 3). Se realizó la verificación de la actividad eléctrica de las Vps izquierdas y derechas observando un bloqueo bidireccional, las maniobras de sobreestimulación no indujeron taquiarritmias atriales. Finalmente, retiramos el catéter de ablación hacia la aurícula derecha y realizamos una línea de ablación en el istmo cavo tricúspideo, alcanzado bloqueo bidireccional de esta zona. El paciente fue dado de alta, con terapia antiarrítmica (propafenona 150 mg tres veces por día y bisoprolol 5 mg una vez al dia) y anticoagulación con warfarina, sin presentar complicaciones agudas ni recurrencias a los 6 meses del seguimiento clínico.

## Discusión

Este es el primer caso reportado de ablación de FA en el Perú en un paciente portador de dispositivo oclusor de comunicación interauricular. Este procedimiento es complejo debido a que la ablación de la FA requiere acceder a la aurícula izquierda a través de una punción transeptal (PST) que puede ser simple o doble, el cual es uno de los pasos críticos de este procedimiento ^4^. Por lo general, la PST se realiza bajo guía ecocardiografía y fluoroscópica ^2^. Sin embargo, la PST en pacientes con dispositivos de oclusión del SIA puede ser un desafío por la visualización limitada o nula del SIA, con un riesgo potencial de complicaciones como perforación en la pared posterior de la aurícula derecha o la raíz aórtica, si la PST se realiza anterior o posterior, respectivamente ^3^^,^^4^. También se han descrito otras complicaciones como el desplazamiento o deformación del dispositivo, punción inadvertida del epicardio, o cortocircuito residual interatrial, que se han reportado como poco frecuentes ^4^^,^^5^. Así mismo se han descrito complicaciones más severas como derrame pericárdico y taponamiento cardiaco ^(^^6^.

La decisión del lugar de punción en el SIA en este paciente con dispositivo oclusor se tomó basado en los hallazgos de la tomografía con reconstrucción 3D realizada antes del procedimiento y con la EIC durante el procedimiento. Si hay suficiente espacio alrededor del dispositivo es posible realizar la PST en el SIA remanente, de lo contrario, es necesario el acceso directo a través del dispositivo, sobre todo en dispositivos ≥ 32 mm o incluso ≥26 mm, de acuerdo con la experiencia del centro ^3^. Un estudio descriptivo realizado en China encontró que en la mayoría de los pacientes la PST fue realizada a través del tabique nativo en la región posteroinferior al dispositivo, en tanto que en pacientes sin espacio de punción se hizo en la cintura del dispositivo ^3^. 

Una revisión sistemática sobre la seguridad y eficacia de la ablación con catéter para la FA en pacientes con dispositivo de cierre del SIA, demostró el éxito de la PST guiado por EIC y fluoroscopia en el 98,4% de los casos revisados, y la no recurrencia de FA en el 77% de los casos durante el seguimiento (6-22 meses) ^4^. Esta revisión también demostró que no hubo diferencias en la recurrencia de FA en pacientes en quienes se realizó la PST a nivel del SIA remanente versus la punción del dispositivo ^4^. En nuestro caso se realizó la técnica de una sola PST, en lugar de la doble punción usual, reduciendo el riesgo de perforación, menor compresión del dispositivo, y menor interposición de los catéteres, los cuales dificultan la reconstrucción 3D y ablación de las VP ^4^.

La EIC es muy útil para lograr la PST en lugares específicos del SIA ^5^^,^^7^. En este caso, el uso de EIC fue importante para guiar toda la PST a través del tabique nativo remanente. De esta manera, la EIC cumplió un rol fundamental para el entendimiento de la anatomía del SIA remanente y la selección del sitio de punción, permitiendo realizarla de forma segura y a la vez ayudar a detectar tempranamente alguna complicación, las que afortunadamente no se presentaron en este caso.

Este reporte de caso nos resalta la importancia de la multimodalidad en imágenes cardiovasculares para la planificación y realización de ablación de FA en pacientes portadores de dispositivos oclusores del SIA. Entre ellos el uso de la tomografía cardíaca con el fin de delimitar los reparos anatómicos exactos a tener en consideración, para que por medio de la EIC se logre una punción transeptal exitosa y eficaz para llevar a cabo la ablación de la FA.
